# Yield of closed pleural biopsy and cytology in exudative pleural effusion

**DOI:** 10.12669/pjms.322.9613

**Published:** 2016

**Authors:** Faisal Faiyaz Zuberi, Bader Faiyaz Zuberi, Syed Khalid Ali, Sagheer Hussain, Farhana Mumtaz

**Affiliations:** 1 Dr. Faisal Faiyaz Zuberi, FCPS (Medicine), FCPS (Pulmonology). Chest Unit-II, Ojha Institute of Chest Diseases, Dow University of Health Sciences, Karachi, Pakistan; 2 Prof. Bader Faiyaz Zuberi, FCPS. Dow Medical College, Dow University of Health Sciences, Karachi, Pakistan; 3 Dr. Syed Khalid Ali, MCPS. Chest Unit-II, Ojha Institute of Chest Diseases, Dow University of Health Sciences, Karachi, Pakistan; 4 Dr. Sagheer Hussain, MBBS. Chest Unit-II, Ojha Institute of Chest Diseases, Dow University of Health Sciences, Karachi, Pakistan; 5 Dr. Farhana Mumtaz, DTCD. Chest Unit-II, Ojha Institute of Chest Diseases, Dow University of Health Sciences, Karachi, Pakistan

**Keywords:** Abram’s needle, Lung cancer, Pleural Diseases, Sensitivity & Specificity

## Abstract

**Objective:**

To determine diagnostic yield of Closed Pleural Biopsy (CPB) and Cytology in Exudative Pleural Effusion (PE).

**Methods:**

This prospective comparative study was conducted at Chest Unit-II & Medical Unit-IV of Dow University of Health Sciences, Karachi Pakistan from January 2011 till December 2014.

**Results:**

Ninety-four patients with exudative PE were finally included. The mean age (SD) was 44.0 (13.8) years. Overall Specific Diagnosis was reached in 76/94 patients; 46 Tuberculosis PE (TPE) & 30 Malignant PE (MPE). CPB diagnosed all TPE patients alone and 28/30 of MPE. Cytology diagnosed only 10/30 patients of MPE with 8 patients having both CPB & Cytology positive for malignancy whereas in the remaining two cases only Cytology positive. The sensitivity of CPB in detecting TPE and MPE was 93.9% and 82.4% respectively whereas specificity for both was 100%. The diagnostic yield of cytology in detecting MPE is only (33.3%). The diagnostic yield of CPB for TPE and MPE is 100% and 93.3% respectively. The overall specific diagnostic yield of CPB is 78.7%.

**Conclusion:**

CPB is better than pleural fluid cytology alone with the later adding little to diagnostic yield when both combined in distinguishing TPE from MPE, the two main differential of exudative PE in a TB-Endemic country.

## INTRODUCTION

Pleural effusion (PE) is a common condition for referral to outpatient clinics or in-hospital consultations inPakistan.^1^ More than fifty causes are possible for PE and hence to treat this condition successfully requires an accurate diagnosis.^2^ Since the beginning of the 19^th^ century, the primary step in evaluation of PE remains diagnostic thoracocentesis, in particular if it is unilateral.^3^ The pleural fluid obtained can then be used to categorize PE into two differentpathophysiological and hence management entities; Transudate and Exudate.^3^ The leading cause for exudative PE differs geographically withparapneumonic PE being the most common etiology in one area and Tuberculous PE (TPE)and Malignant PE (MPE) in others.^2^,^4^ In approximately 40% of exudative PE, the initial thoracocentesis fail to give the diagnosis and the next step is then Closed Pleural Biopsy (CPB).^3^ Pakistan is an endemic country for Tuberculosis (TB) and the microbiological yield of Acid Fast Bacilli (AFB)in TPE is reported as less than 50%.^3^,^5^ This increases the importance of doing CPB with pleural fluid cytology in exudative PE to differentiate TPE from MPE.

The pleural tissue can be acquired by doing pleural biopsy with the Closed (blind), Image-guided or Thoracoscopic techniques.^2^ The choice depends on factors related to condition of the patient, diagnostic yield, availability of instrument facility, expertise and finally the cost. Thoracoscopy although has greater yield than CPB for the diagnosis of MPE but for TPE does not add to yield over CPB.^6^ Further, thoracoscopy has several restrictions like scarce availability, costly and need for thoracic surgery backup.^6^ All of this contribute in making CPB in our cost constraint country as the first choice for exudative PE evaluation instead of thoracoscopy. CPB was first done by Defrancis et al using Vim Sliverman needle in 1955 andsince then various CPB needles; Abram, Cope, Raja, Ramel, named after their inventors are used.^7^ Abram’s CPB needle constantly remains the most popular with higher diagnostic yield than Cope and Vim Sliverman needle.^8^ Besides, Abram’s needle is easy to use, can be performed at bedside, safe and economic. The diagnostic yield of CPB depends on geographical area, patient selection and the number of pleural tissues taken.^5^ Hence there is a wide variation in its diagnostic yield; 50% to 80% for TPE and 40% to 75% for MPE.^9^ The diagnostic yield of pleural fluid Cytology for MPE shows even more wide range from 40% to 90%.^9^ There is limited data from our country with only two studies on this common and important subject were found on the PubMed search although some studies are available in locally indexed journals.

This study was done to determine diagnostic yield of CPB and cytology in exudative PE. This will help us evaluate two diagnostic modalities and thus will have a positive impact on clinical practice in our area. CPB is an inexpensive and easily learned procedure and its validity will ensure easy, economical and early diagnosis. This will lead to better and early treatment of patients.

## METHODS

This prospective study was carried out in Chest Unit-II of Ojha Institute of Chest Diseases and Medical Unit-IV Civil Hospital Karachi, both arePostgraduate Tertiary Care Hospitals affiliated with Dow University of Health Sciences Karachi, Pakistan from January 2011 till December 2014 for a period of four years. Study has approval of Institutional Review Board of Dow University of Health Sciences vide letter # IRB-670/DUHS/Approval/2015/135. Proforma included fields for routine basic investigations, sputum AFB smear x2 and chest radiographic findings. Data was also recorded for Pleural fluid DR, Gram’s stain, Routine Culture and Sensitivity (CS), AFB smear and cytology. Only those found to have exudate type of PE (pleural fluid protein > 3.5g/dl) with minimum of 50 mL of pleural fluid sent for Cytology were included in the study.^10^ Patients with Pleural fluid gram stain or routine CS positive were excluded along with those having AFB smear positive in pleural fluid or sputum. Data was also recorded for the result of percutaneous CPB that was done for initial non-diagnostic result of investigations for exudative PE. CPB was performed with Abram’s needle having at least six pleural tissue specimen by Pulmonology postgraduate trainee or faculty were included. TPE was diagnosed when CPB reveled on histopathology caseating granulomas whereas MPE with malignant pattern on pleural biopsy or malignant cells detected in pleural fluid cytology. In cases where CPB did not reveal definite diagnosis, further investigations were also conducted to reach specific diagnosis.

Mean age with Standard Deviation(SD) of selected patients was calculated and compared among groups based on results of CPB by ANOVA. Pleural fluid cytology findings were also compared by ANOVA. Sensitivity and Specificity and both positive and negative predictive values for CPB were calculated by using a decision matrix.^11^

## RESULTS

**Fig.1:**
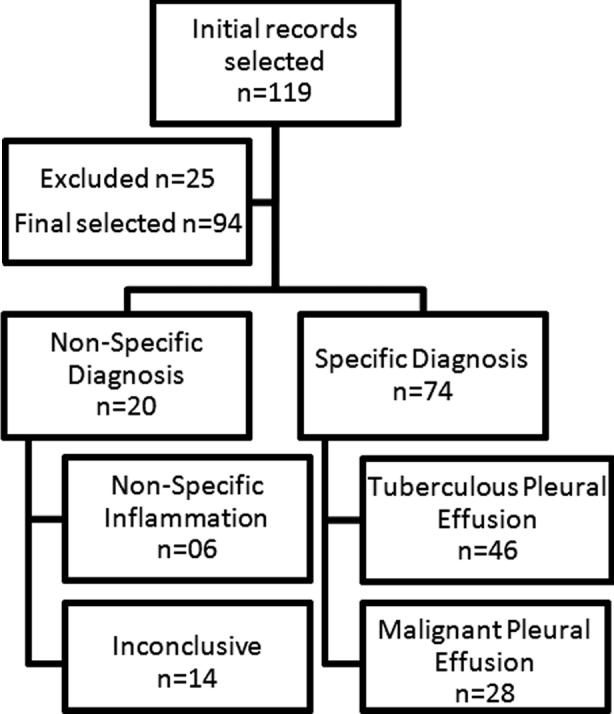
Results of closed pleural biopsy.

A total of 94 patients meeting inclusion criteria were selected. The mean age (±SD) was 44.0 (13.8) years. Overall Specific Diagnosis was reached in 76/94 patients (46 TPE & 30 MPE). CPB diagnosed all TPE patients alone and 28/30 of MPE respectively. Cytology diagnosed only 10/30 patients of MPE with 8 patients having both CPB & Cytology positive for malignancy whereas in the remaining two cases CPB was negative but Cytology positive. As shown in Fig.1, CPB gave Specific Diagnosis in 74 (78.7%) patients and the remaining 20 (21.2%) cases had Non-Specific Diagnosis. On further workup of this later group of patients revealed the following diagnosis: MPE (06), TPE (03), Rheumatoid Arthritis (02), Hyperthyroidism (02), Systemic lupus erythematosus (01), Hydatid (01), Unknown etiology (03) and (02) cases workup was not complete to reach the specific diagnosis.

CPB revealed four patterns; Pleural Tuberculosis (46/94, 48.9%), Malignancy (28/94, 29.8%), Nonspecific Inflammation (06/94, 6.4%) and Inconclusive (14/94, 14.9%). The mean age (SD) according to the patterns of CPB was 40.9 (13.8) years in TB, 48.1 (13.0) years in Malignancy, 44.0 (13.5) years in Nonspecific Inflammation and 45.9 (14.2) years in Inconclusive. Testing for difference in age according to pattern of CPB using ANOVA within group analysis, we did not find any significant difference (df = 90; p = 0.166).

Pleural fluid Cytology had only two groups; Non Malignant (84/94, 89.4%) and Malignant (10/94, 10.6%). Analysis of mean age (SD) according to groups on cytology was 43.3 (13.4) years in nonmalignant group and 50.0 (16.0) years in malignant group. No significant difference was observed on within group ANOVA analysis (df = 92; p = 0.144).

Out of 28 patients confirmed with Malignancy on CPB; only 08 (28.6%) showed malignant cells on Pleural Fluid Cytology whereas in 20 (71.4%) patients with malignancy on CPB, Cytology was negative. Cytology of all the patients diagnosed on CPB as having Pleural Tuberculosis was negative as well. Six patients who had Nonspecific Inflammation on CPB, Cytology also showed no malignant cells whereas 14 patients whose CPB was Inconclusive, two showed malignant cells on Cytology. Details are given in Table-I. Significant difference was observed in Pleural fluid Cytology findings among the patterns of CPB (p= 0.001).

**Table-I:** Cross tabulation of findings of pleural biopsy with pleural fluid cytology.

Pleural Biopsy	Cytology		Total

	Non Malignant	Malignant	
Inconclusive	12	2	14
	85.7%	14.3%	100.0%
Malignant	20	8	28
	71.4%	28.6%	100.0%
Pleural TB	46	0	46
	100.0%	0.0%	100.0%
Nonspecific Inflammation	6	0	6
	100.0%	0.0%	100.0%
Total	84	10	94
	89.4%	10.6%	100.0%

P = 0.001

To analyze the cumulative effect of nonmalignant patterns on CPB together along with their malignant component with that of the respective nonmalignant and malignant groups based on pleural fluid cytology; all the nonmalignant patterns (Pleural tuberculosis, Inconclusive and Nonspecific Inflammation) of CPB were merged as one group and analyzed for significance with malignant and nonmalignant findings on pleural fluid cytology using 2x2 table. Significant difference was observed in the malignant and nonmalignant groups of CPB with those of the Pleural Fluid Cytology respectively (p=0.001).

The Malignant group based on CPB (n=28) revealed the following histopathological diagnosis:Metastatic Adenocarcinoma (23/28), Mesothelioma (03/28), Squamous Cell Carcinoma (01/28) and B-Cell Lymphoproliferative Disorder (01/28). In two patients in which CPB was negative for malignancy (the combined nonmalignant group), the Pleural fluid Cytology showed malignant cells. In both of these later cases Metastatic Adenocarcinoma was detected.

In total of twenty-five patients with the diagnosis of metastatic adenocarcinoma, the primary origin of malignancy suggested by the Histopathologist was as follows: Lung (11/25), Breast (04/25), Ovarian Carcinoma (02/25), Gastrointestinal (02/25), Renal (01/25), Thyroid (01/25) and in the remaining four cases primary could not be ascertained.

The sensitivity of CPB in detecting TPE and MPE was 93.9% and 82.4% respectively whereas specificity for both was 100%. Table-III. The diagnostic yield of cytology in detecting MPE is only (10/30,33.3%). The diagnostic yield of CPB for TPE and MPE is 100% and 93.3% respectively. The overall specific diagnostic yield of CPB is 78.7%.

**Table-II:** Cross tabulation of Merged Pleural Biopsy patterns with Pleural Fluid Cytology.

Pleural Biopsy	Cytology		Total

	Non Malignant	Malignant	
Non Malignant	64	2	66
	97.0%	3.0%	100.0%
Malignant	20	8	28
	71.4%	28.6%	100.0%
Total	84	10	94
	89.4%	10.6%	100.0%

P= 0.001

**Table-III:** Diagnostic Statistics of Closed Pleural Biopsy*.

Parameter	TPE		MPE	

	Present	Absent	Present	Absent
Positive	46	0	28	0
Negative	3	43	6	58
Sensitivity (%)	93.9%	82.4%		
Specificity (%)	100%	100%		
PPV (%)	100%	100%		
NPV (%)	93.5%	90.6%		

* Two cases were excluded from analysis for incomplete workup

TPE: Tuberculous Pleural Effusion,MPE: Malignant Pleural Effusion,

PPV: Positive Predictive Value,NPV: Negative Predictive Value.

## DISCUSSION

Although some studies on the subject have been published in Pakistani journals; this is the largest study from Pakistan in internationally indexed PubMed Journal with 94 patients; the two others from our country reported on 68 & 63 patients only.^1^,^5^ The mean age in our study is higher than that reported by HS Hira et al. (44.0 vs 31.7 years) and lower than several studies by others (44.0 vs 72.0-48.0 years).^5^,^10^,^12^-^16^ In our study the patients diagnosed with MPE were older than those with TPE in both the groups of CPB (mean age 48.1 vs 40.9 years) and Cytology (mean age 50.0 vs 43.3 years). This is consistent with other studies.^9^, ^15^ Besides, the difference in age was not statistically significant in our study, emphasizing that age alone cannot reliably differentiate between TPE and MPE. This has been reported that although MPE is more common in patients aged over 60 years but reactivation of TB can also present as TPE in this older age group.^15^

The sensitivity of Closed CPB for detecting TPE and MPE in our study is higher than that reported in various studies (93.9% vs 57%-80% for TPE) and (82.4% vs 40%-73% for MPE) respectively.^17^,^18^ The positive predictive value was 100% for both (TPE & MPE) in our study which is consistent with the study done by Al-Shimemeri et al. from Saudi Arabia.^13^ Moreover, that study had a lower negative predictive value than that for our study for both TPE (76.1% vs 93.5%) and MPE (84.8% vs 90.6%) respectively.

Our study showed the overall diagnostic yield of CPB was 78.7% which is lower than that reported by Ihsanullah et al.(78.7% vs 95%) and higher than that published by Hussain SF et al. (78.7% vs 46%), both studies are from Pakistan.^1^,^5^ The lower yield in the later study can be due to inclusion of patients with PE of transudate, unknown protein content and with liver disease and heart failure. On comparison with studies from abroad, the diagnostic yield of our study was similar on one hand (78.7% vs 76% &84.5%) and higher with a wide difference on the other.(78.7% vs 49.1% & 59.6%).^13^,^16^,^19^ Our results are consistent with two other studies from TB-Endemic areas in having TPE as most common cause for exudative PE (48.9% vs 44.1% & 43.7%)and MPE being found in (31.9% vs 32.1% & 29.6%) of patients.^4^,^20^,^21^

As shown in Table-I when different diagnostic patterns was observed on CPB result were analyzed with that of based on Pleural Fluid Cytology, significant difference was observed implying CPB is better than Cytology in reaching the diagnosis. However, this difference of higher overall diagnostic yield of CPB over Cytology (78.7% vs 33.3%) could be attributed to larger number of patients diagnosed with TPE than with MPE in our study (n=46 vs 30)and lack of role of Cytology in contributing to diagnosis of TPE. To take care of this, we merged the patterns on CPB to have two groups, Malignant and Non Malignant, and analyzed with the same labelled groups based on Cytology. CPB again emerged as statistically superior to Cytology as shown in Table-II. This higher diagnostic yield and sensitivity of CPB for MPE alone 93.3% and 82.4% respectively is in contrast to other studies which have reported CPB sensitivity of only 48%-56%.^3^,^22^,^23^ Notwithstanding this, studies have shown 7%-12% of MPE can be diagnosed by CPB when cytology is negative.^12^ The diagnostic yield of Cytology is affected by the type of primary tumor, the proficiency of the cytopathologist, number and amount of the specimen.^14^ One study showing 60 mL of pleural fluid for Cytology has better yield than 10 mL whereas the other reporting that greater than 50 mL did not enhance the yield.^24^ Furthermore, sending more than two samples of cytology does not add significantly to the yield (First sample 65%, Second 27% and Third only 5%).^25^

One explanation for this higher diagnostic yield in our study of CPB over Cytology for detecting MPE is that being a resource-limited country with CPB facility available in a few centers, patients present late with advanced disease implicating more extensive involvement of pleura by malignancy. Besides, the inclusion of patients in our study with clear cut exudative PE, thereby excluding borderline cases which would have decreased the yield, and at least six pleural biopsy tissue specimens, altogether these augment the diagnostic yield.^8^,^10^

## CONCULSION

CPB is better than pleural fluid cytology alone with the latter adding little to diagnostic yield when both combined in distinguishing TPE from MPE, the two main differential of exudative PE in a TB-Endemic country with limited resources.
